# Research advances in probiotic fermentation of Chinese herbal medicines

**DOI:** 10.1002/imt2.93

**Published:** 2023-02-19

**Authors:** Xiaoling Zhang, Qin Miao, Chengxue Pan, Jia Yin, Leli Wang, Lingbo Qu, Yulong Yin, Yongjun Wei

**Affiliations:** ^1^ School of Pharmaceutical Sciences, Key Laboratory of Advanced Drug Preparation Technologies, Ministry of Education Zhengzhou University Zhengzhou China; ^2^ Laboratory of Synthetic Biology, Food Laboratory of Zhongyuan Zhengzhou University Zhengzhou China; ^3^ Hunan Provincial Key Laboratory of Animal Intestinal Function and Regulation, College of Life Science Hunan Normal University Changsha China; ^4^ College of Chemistry Zhengzhou University Zhengzhou China; ^5^ Institute of Subtropical Agriculture Chinese Academy of Sciences Changsha China; ^6^ Jiangsu Collaborative Innovation Center of Chinese Medicinal Resources Industrialization Nanjing University of Chinese Medicine Nanjing China

**Keywords:** Chinese herbal medicine, probiotics, fermentation, microbiome, synthetic biology

## Abstract

Chinese herbal medicines (CHM) have been used to cure diseases for thousands of years. However, the bioactive ingredients of CHM are complex, and some CHM natural products cannot be directly absorbed by humans and animals. Moreover, the contents of most bioactive ingredients in CHM are low, and some natural products are toxic to humans and animals. Fermentation of CHM could enhance CHM bioactivities and decrease the potential toxicities. The compositions and functions of the microorganisms play essential roles in CHM fermentation, which can affect the fermentation metabolites and pharmaceutical activities of the final fermentation products. During CHM fermentation, probiotics not only increase the contents of bioactive natural products, but also are beneficial for the host gut microbiota and immune system. This review summarizes the advantages of fermentation of CHM using probiotics, fermentation techniques, probiotic strains, and future development for CHM fermentation. Cutting‐edge microbiome and synthetic biology tools would harness microbial cell factories to produce large amounts of bioactive natural products derived from CHM with low‐cost, which would help speed up modern CHM biomanufacturing.

## Highlights

- Fermentation of Chinese herbal medicines (CHM) using probiotics can generate easily absorbed bioactive substances and reduce toxicities.
- Probiotic fermentation techniques for CHM are described and discussed.
- Microbiome, synthetic biology, and other cutting‐edge biotechnologies improve probiotic fermentation of CHM.

## Graphical abstract

This review summarizes the advantages of probiotic fermentation of Chinese herbal medicine (CHM), fermentation techniques, probiotic strains, and future development of CHM fermentation. Besides, synthetic biology would harness microbial cell factories to de novo produce large amounts of CHM bioactive natural products with low‐cost, and it might be an opportunity to bring CHM into modern biomanufacturing.

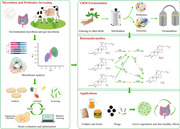

## INTRODUCTION

Traditional Chinese medicine is one of the oldest healing systems, including herbal medicines, acupuncture, moxibustion, massage, food therapy, and a few other therapeutic strategies [1]. Chinese herbal medicines (CHM) refer to natural medicines and their processed products, and are mainly composed with plant medicines (including root, stem, leaf, and fruit) and mineral medicines [2]. Most CHM are derived from medicinal plants, and they have been used to treat human diseases in China and other Asian countries for thousands of years [3]. CHM contain hundreds of different components with diverse physiochemical properties based on metabonomic analysis [4, 5]. The artemisinin, one bioactive compound extracted from *Artemisia annua*, has been used for the treatment of malaria and other diseases [6, 7]. In the 3 years from 2018 to 2020, artemisinin‐based combination therapies had been used to treat more than 454 million malaria cases. Besides, some classical Chinese medicinal prescriptions based on CHM have been applied for the treatment of anxiety, insomnia, cognitive impairment, and other diverse difficult diseases [8].

The contents of some bioactive ingredients in CHM are lower than 1% [9, 10, 11, 12], and some CHM components are toxic to humans and animals [13, 14]. Microbial fermentation is one of the traditional CHM processing techniques, which reacts under proper temperature, humidity, and moisture conditions [15, 16]. CHM fermentation could increase pharmaceutical efficacy, reduce toxicity, produce new chemical components, and protect wild herb resources [15, 17]. The records of fermented CHM and its products were available in “Qi Min Yao Shu,” “Shen Nong Ben Cao Jing,” “Ben Cao Gang Mu,” and “Pharmacopoeia of the People's Republic of China,” including Pinelliae Rhizoma Qu, Shen Qu, Jian Shen Qu, Cai Yun Qu, Chen Xiang Qu, Semen Sojae Praeparatum, Bai Yao Jian, and Pien Tze Huang [15]. Moreover, some fermented CHM have been applied in animal feeding, and they are demonstrated to be beneficial for animal health. For example, Massa Medicata Fermentata (Shenqu or Liushenqu) improves intestinal homeostasis during piglets weaning [18], and probiotics‐fermented herbal blend can improve the growth performance of *Salmonella pullorum*‐infected chicks [19].

Normally, chemical compositions and contents of CHM were changed after microbial fermentation. Some effective ingredients of CHM can only be transformed and absorbed by the gut microbiota [17]. As the gut microbiota composition of hosts and their drug absorption capacity are personalized [20], in vitro fermentation could standardize CHM products and enhance the clinical efficacy of CHM [21, 22, 23]. Actually, a few fermented CHM have better pharmacological activity than the nonfermented CHM [15, 16]. Probiotics are live microorganisms that have demonstrated beneficial effects on human health [24]. Both probiotics and some CHM are beneficial for human gut microbes [25], intestinal epithelial barrier [26], and immune system [27], thus, fermentation of some CHM with probiotics is of great interest.

Some clinical trials on the use of probiotics‐fermented CHM showed promising clinical effects. Fermented milk containing *Lactobacillus paracasei* and the CHM *Glycyrrhiza glabra* is beneficial for patients infected with *Helicobacter pylori*; the treatment group significantly improved gastrointestinal symptoms and quality of life, and no serious adverse events were observed [28]. An open‐label, randomized, single‐dose, two‐period, and crossover study of the main ginsenoside metabolites, compound K, was conducted in 12 Japanese healthy subjects, showing that the absorption of compound K increased significantly after the intake of fermented ginseng compared with nonfermented ginseng [29]. Additionally, ginseng fermented by *L. paracasei* A221 improved the first‐night effect in humans [30]. Fermented red ginseng lowered postprandial glucose levels in subjects with impaired fasting glucose or type 2 diabetes [31], and improved nasal congestion symptoms and quality of life in patients with perennial allergic rhinitis [32].

Though CHM fermentation has been applied to herbal drug preparation, the underlying biotransformation mechanisms of most CHM fermentation are unclear. Therefore, global and systematic analyses of CHM fermentation are necessary. In this review, we focus on the summary and discussion of current probiotic fermentation of CHM, including the potential mechanisms of CHM fermentation, the CHM fermentation advantages, the probiotics used for CHM fermentation, and modern microbial fermentation technologies. Moreover, future microbiome strategies for CHM fermentation using probiotics and the application of synthetic biology in the production of CHM bioactive ingredients are discussed.

## MECHANISMS OF CHM FERMENTATION BY PROBIOTICS

Compared with traditional CHM processing methods, fermentation of CHM with probiotics can improve CHM bioactivity under mild processing conditions [33]. First, some CHM natural products are difficult to absorb and utilize in vivo. In the meanwhile, several hydrolases produced by probiotics during CHM fermentation can destroy plant cell walls and promote the release of bioactive ingredients in CHM [34]. *Streptococcus lactis* could efficiently degrade the cellulose, and the fermentation of Astragalus with *S. lactis* increased the contents of crude polysaccharides, total flavonoids, and total saponins in Astragalus roots, stems, and leaves [35]. Secondly, most herbal medicines are orally administered to humans, and CHM components can be transformed by gut microbiota before absorption [20, 36]. The enzymes secreted by gut probiotics can hydrolyze and remove glycosyl groups from CHM natural products, which increases their lipophilicity and improves the absorption rate in the gastrointestinal tract. After oral ingestion of liquorice, glycyrrhizin is converted to glycyrrhizic acid, and subsequently converted to glycyrrhetinic acid by gut microbiota [37]. In addition, probiotic fermentation can reduce or degrade the toxicity of some CHM [38].

**Figure 1:**
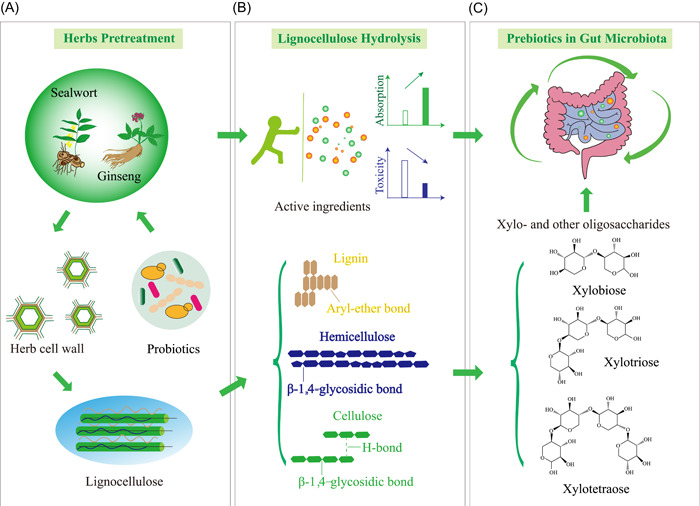
Lignocellulases and their functions in sealwort, ginseng, and other Chinese herbal medicine (CHM) fermentation. (A) The lignocellulose might prevent the release of bioactive ingredients of CHM, and lignocellulases derived from probiotics or other microbes can be used to degrade herb lignocellulose. (B) Lignocellulose hydrolysis releases bioactive ingredients in herbs, and leads to the generation of oligosaccharides prebiotics. (C) Bioactive ingredients and oligosaccharides are beneficial for the gut microbiota of humans and animals.

Some effective natural products in CHM can be acted as prebiotics, which promote the proliferation of beneficial microorganisms in hosts [39]. The intake of yam significantly changed mice' gut microbiota, and the numbers of Bifidobacterium and Lactobacillus increased in mice [40]. Astragalus, Angelica, *cowherb seed*, Codonopsis, Licorice, and *ligustici wallichii* could individually stimulate the proliferation of probiotics, such as *Bacillus subtilis*, *Lactobacillus acidophilus* and yeasts, in a dose‐related manner [41]. Both red ginseng and *Semen Coicis* promoted the growth of Bifidobacterium and Lactobacillus in vitro, and improved the gut microbiota and relieved the symptoms of ulcerative colitis in vivo (Figure 1) [42]. *Flos lonicerae* has a significant regulatory effect on gut dysbiosis of mice, which could promote the recovery of gut microbiota dysbiosis [43]. Thus, the synergistic effect of CHM fermented with probiotics might enhance the effectiveness of CHM.

## ADVANTAGES OF PROBIOTIC FERMENTATION OF CHM

### Promoting the release of effective ingredients and improving the pharmacological activities of CHM

The effective ingredients of CHM are mostly distributed in the cytoplasm of root, stem, and leaf cells of plant biomass. The plant cell wall structure is tight, and is mainly composed of cellulose, hemicellulose, and lignin, which hinders the release of bioactive natural products and results in low absorption and utilization of CHM bioactive natural products [44]. Probiotics can produce a variety of hydrolytic enzymes, especially lignocellulases, to degrade plant cell wall and promote the release of bioactive natural products in CHM [45, 46] (Figure 1A,B). These released bioactive natural products include flavonoids, glycosides, anthraquinones, terpenoids, alkaloids, and organic acids (Table 1). Moreover, the lignocellulases can help generate oligosaccharide prebiotics for the gut microbiota of humans and animals (Figure 1C). Therefore, probiotic fermentation can improve the pharmacological activity of CHM [47].

**Table 1:** Contents of effective Chinese herbal medicine ingredients increased after microbial fermentation.

Herbs/herb formula	Microorganism	Increased bioactive natural products	Pharmacological effects	References
Hwangryun‐haedok‐tang	*Lactobacillus curvatus*	Baicalin	Ovariectomy‐induced bone loss ↓	[48]
*Condonpsis lanceolata*	*Bifidobacterium longum, Lactobacillus acidophilus, Leuconostoc mesenteroides*	Gallic acid and vanillic acid	Neuroprotective effect ↑ Cognitive enhancing activity ↑	[49]
*Artemisia princeps* Pampanini	*Lactobacillus plantarum*	Catechol and seco‐tanapartholide C	Anti‐inflammatory activity ↑	[50]
*Panax notoginseng*	*Lactobacillus helveticus, Lactobacillus rhamnosus, L. acidophilus*	Ginsenoside Rg3 and Rh1	Anti‐hepatocarcinoma activity ↑	[51]
*Astragalus membranaceus*	*Enterococcus faecium*, *L. plantarum*	Astragalus polysaccharide, total saponins, and flavonoids	Not determined	[52]
*Polygonum cuspidatum*	*Aspergillus niger*, yeast	Resveratrol	Not determined	[53]
*Radix astragali*	*Aspergillus oryzae*	3,4‐Di(4′‐hydroxyphenyl) isobutyric acid	Antioxidant activity ↑	[54]
Red ginseng (the steamed ginseng)	*Phellinus linteus*	Ginsenosides Rg3, Rg5, Rk1, compound K, Rh1, F2, and Rg2	Skin permeability ↑	[55]
	*Lactobacillus brevis*	Ginsenosides Rg3, Rg5, Rk1, compound K, Rh1, F2, Rg2, and flavonoids	Antiwrinkle efficacy ↑ Skin sensitization ↓	[56]
	*L. plantarum*	Ginsenoside Rd and total phenolic	Antioxidant activities ↑	[57]
	*Lactobacillus paracasei, B. longum*	Ginsenosides Rg3, F2, Rh1, Rh2, and Rg2	Ovalbumin‐induced inflammation ↓	[58]
*Panax ginseng*	*Ganoderma lucidum* mycelium	Polysaccharides	Immunological activity ↑	[59]
	*Lactobacillus fermentum*	Rare ginsenosides (Rg2, Rg3, Rh1, Rh2, F2, and Ro)	Hyperlipidemia ↓ liver injury ↓	[60]
*Dendrobium officinale*	*Bacillus* sp. DU‐106	Polysaccharides with high proportion of mannose	Immunoregulatory activities ↑	[61]

After fermentation of CHM with the probiotics, such as *Lactobacillus casei*, *Enterococcus faecalis*, and *Candida utilis*, the contents of soluble total flavonoids, total alkaloids, crude polysaccharides, and total saponins in the fermented Chinese herbs of *Semen vaccariae* and *Leonurus artemisia* increased by 55.14%, 127.28%, 55.42%, and 49.21%, respectively, compared with the natural herbs [62]. After fermented by *Lactobacillus pentosus*, the contents of quercetin and kaempferol in the extracts of *Lespedeza cuneata* G. Don increased by 242.9% and 266.7%, respectively, which improved potential antioxidative and antiaging functions of the herb [63]. After fermentation with *Bifidobactericum breve* strain CCRC 14061, the contents of daidzein and genistein in Puerariae Radix increased 785% and 1010%, respectively, which can stimulate hyaluronic acid production in NHEK cells [64]. Fermenting *Cordyceps militaris* with *Pediococcus pentosaceus* (GRC‐ON89A) enhanced phagocytic activity of RAW 264.7 cells and primary cultured murine macrophages; the enhanced immune activity of *C. militaris* was attributed to the increased content of *β*‐glucan, cordycepin, and short‐chain fatty acids after fermentation [65]. The microbial fermentation, especially probiotic fermentation, can significantly increase the contents of bioactive natural products and improve the pharmacological effects of CHM (Table 1).

### Reducing toxicities and side effects of CHM

Some CHM have certain toxicities to humans and animals, and direct oral intake of them would generate serious toxic effects [66]. Probiotics can degrade or modify the toxic components, thus, reduce the toxicities or side effects of CHM [67, 68]. Conjugated anthraquinones are the main components leading to severe diarrhea of rhubarb. Fermentation of rhubarb with *Kluyveromyces marxianus* KM12 could convert conjugated anthraquinone to free anthraquinone, and the side effects of severe diarrhea generated by rhubarb were alleviated [69]. Compared with the original crude *Croton tiglium*, fermentation of *C. tiglium* with *Ganoderma lucidum* and *Beauveria bassiana* could decrease acute oral toxicity by about four times, and have no inflammation effect and hemocytolysis [70]. Fermentation of *Tripterygium wilfordii* with *G. lucidum* reduced the hepatotoxicity of *T. wilfordii*, which was due to the decrease of wilforlide after fermentation [71].

### Generating new bioactive substances and enhancing the bioavailability of CHM

**Figure 2:**
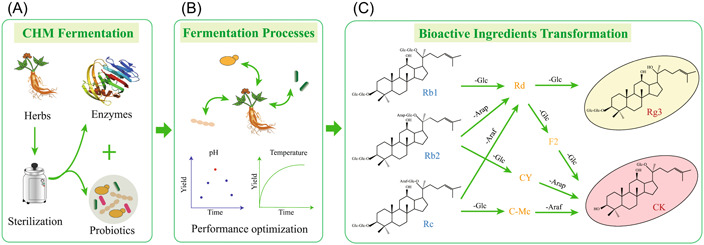
Biotransformation of ginsenosides to active rare ginsenosides using efficient enzymes or probiotics. (A) The herbs of *Panax ginseng* are sterilized for probiotic fermentation, and the enzymes and probiotics are the main driving forces for CHM fermentation. (B) Probiotic performance during ginseng fermentation can be optimized to improve bioactive ingredient yield. (C) Ginsenosides can be transformed to the bioactive rare ginsenosides during CHM fermentation. CHM, Chinese herbal medicines.

Probiotic fermentation transforms CHM ingredients to new bioactive compounds, and this might bring new pharmacological characteristics to CHM (Figure 2). Ginsenosides are the main physiologically bioactive natural products of ginseng, and ginsenosides Rb1, Rb2, Rc, Re, and Rg1 constitute more than 80% of the total ginsenosides in *Panax ginseng* [72]. Some rare ginsenosides, such as F2 and Rd, are demonstrated to have high bioavailability and bioactivity. However, their contents in natural *P. ginseng* are extremely low, and some of them, such as compound K (CK), are not available until *P. ginseng* biomass is transformed in human body [72]. The probiotic, *Bifidobacterium animalis* subsp. *lactis* LT 19‐2, can effectively convert main ginsenosides Rb2 and Rb3 in red ginseng extracts to rare ginsenosides of Rd, Rh1, F2, and Rg3 (Figure 2C) [73]. Probiotic fermentation of red ginseng increased RAW 264.7 cells macrophage activity significantly and activated primary immune cells, including splenic cells and bone marrow‐derived macrophages, suggesting that fermentation with *B. animalis* subsp. *lactis* LT 19‐2 can improve the immunomodulatory function of red ginseng [73]. The probiotics‐fermented red ginseng significantly increased Th1 and Treg cell differentiation, which could activate macrophages in mice to alleviate cyclophosphamide‐induced immunosuppression and 2,4,6‐trinitrobenzenesulfonic acid‐induced colitis [74]. The probiotics‐fermented herbal blend can enhance the immune ability of chicks infected with *S. pullorum* [19]. In another study, fermentation of *P. ginseng* extracts with *B. lactis* and *Lactobacillus rhamnosus* HN001 transformed ginsenosides of Rb1, Rc, and Rb2 to Rd (Figure 2B,C) [75]. The probiotics fermentation or enzymatic catalysis can generate bioactive rare ginsenosides (Figure 2). Fermentation of *Dioscorea opposita* Thunb. with *Saccharomyces boulardii* generates a series of novel low‐molecular‐weight polysaccharides, and these polysaccharides are easy to digest and have improved antioxidant activity and radioprotection effects [76]. Probiotics enabled the production of novel bioactive substances during fermentation. Further insights into the functional mechanisms of probiotics‐fermented CHM would pave the way to rational design of proper fermentation strategies.

### Reducing production costs and protecting environments

Probiotic CHM fermentation can increase the contents of effective ingredients and decrease the consumption of CHM. Many natural CHM resources, such as wild Panax and Glycyrrhiza resources, decreased in the past few years [60, 77]. Rare ginsenosides have been used to produce anticancer drugs, foods, and health care products [78], and probiotic fermentation could reduce the consumption of *P. ginseng* and the production costs of rare ginsenosides.

During CHM processing, large amounts of CHM residues were generated, direct abandonment or incineration of the residues would waste resources and generate environmental pollutions [79]. Huazhenghuisheng oral liquid (HOL), a clinical anti‐lung and liver cancer drug, is produced with 35 kinds of CHM. Fermentation of HOL residues with *Aspergillus cristatus* CB10002 could produce valuable compounds of anthraquinones [80]. The *Lactobacillus plantarum* HM218749 was used to ferment herb residues generated during the production of Jianweixiaoshi tablets, and the fermentation supernatant showed strong anti‐*H. pylori* activity in mice [81]. The fermented residues of one CHM formula composed of Pulsatilla, Rhizoma Coptidis, Cortex Phellodendri, Cortex Fraxini, Rhizoma Atractylodis, Rhizoma *Artactylodis macrocephalae*, and Granati Pericarpium, could improve the antioxidant capacity and immunity in weaned piglets, showing the fermented residues have potentials to be used as substitutes for antibiotics in piglets' feeding [82]. Probiotic fermentation can help reduce CHM consumption and provide a green recycling strategy of herb residues, which would save natural CHM sources, reduce production costs, and protect environments.

## PROBIOTICS COMMONLY USED IN CHM FERMENTATION

A total of 35 species or subspecies microbes have been approved in China as edible probiotics [17], and some of them have been used to ferment CHM (Table 2). Lactobacillus is the most used probiotic genus in CHM fermentation. Lactobacillus has been used to ferment *P. ginseng* [21], Rhizoma *A. macrocephalae* [83], *Anoectochilus formosanus* Hayata [84], *L. cuneata* G. Don [63], Danshen [85], and some herb formulas, including Soshiho‐tang [86], Jaeumganghwa‐tang [87], and Hwangryun‐haedok‐tang [88]. Bifidobacterium species have been used to ferment Radix Puerariae [64] and *A. formosanus* Hayata [84]. Bacillus species have been used to ferment Danshen [85], ginseng seed [89], and Rhizoma *A. macrocephalae* (Table 2) [90]. Some fungi, especially medicinal fungi, have been applied in CHM or herb formulas fermentation. Saccharomyces have been used to ferment *Glycyrrhiza uralensis* Fisch [91] and *Gegen Qinlian decoction* [92]; and *G. lucidum* has been used to ferment *Artemisia capillaris* leaves [93] (Table 2). Currently, most CHM fermentation is still limited to a single‐strain fermentation of single Chinese herb, and few studies on fermentation of CHM with multiple probiotics or synthetic microbiota were reported [19, 94, 95]. As diverse probiotics or probiotic combinations are available in nature, screening novel probiotic strains or building synthetic probiotic microbiota might improve CHM fermentation [96, 97].

**Table 2:** List of probiotics, medicinal fungi, and a few industrial fungi used for Chinese herbal medicine fermentation.

Category	Genus	Species	Herbs/Herb formulas used for fermentation	References
Bacteria	Lactobacillus	*L. plantarum*	Red ginseng; Jianweixiaoshi tablets; Soshiho‐tang; Rhizoma *Artactylodis macrocephalae*	[57, 81, 83, 86]
		*L. acidophilus*	*Anoectochilus formosanus* Hayata; Jaeumganghwa‐tang	[84, 87]
		*L. casei*	*A. formosanus* Hayata; Hwangryun‐haedok‐tang	[84, 88]
		*L. paracasei*	Red ginseng	[98]
		*L. pentosus*	*Lespedeza cuneata* G. Don	[63]
		*L. rhamnosus*	*Panax ginseng*; *Salvia miltiorrhiza* Bunge	[75, 85]
		*L. gasseri*	Ginseng seed	[89]
		*L. fermentum*	*P. ginseng*	[21]
	Bifidobacterium	*B. breve*	Radix Puerariae	[64]
		*B. longum*	*A. formosanus* Hayata; Red ginseng	[84, 98]
		*B. lactis*	*P. ginseng*	[75]
		*B. animalis* subsp. *lactis*	Red ginseng	[73]
	Bacillus	*B. subtilis*	*S. miltiorrhiza* Bunge; Ginseng seed; Deer antler; White ginseng roots	[85, 89, 99, 100]
		*B. licheniformis*	Rhizoma *A. macrocephalae*	[90]
	Alcaligenes	*A. spiechaudii*	*Rhodiola rosea*; *Lonicera japonica*	[101]
	Lactococcus	*L. lactis*	*P. ginseng*	[98]
	Streptococcus	*S. thermophiles*	*Cyclopia intermedia*	[102]
	Leuconostoc	*L. mesenteroides*	*R. coptidis*	[103]
	Pediococcus	*P. pentosaceus*	Ginseng seed	[89]
Fungi	Saccharomyces	*S. cerevisiae*	*Glycyrrhiza uralensis* Fisch; *Gegen Qinlian decoction*	[91, 92]
		*S. boulardii*	*Dioscorea opposita* Thunb	[76]
	Kluyveromyces	*K. marxianus*	*Rhubarb*	[69]
	Trichoderma	*T. reesei*	White ginseng roots	[100]
	Ganoderma	*G. lucidum*	*Croton tiglium*; *Tripterygium wilfordii*; *Artemisia capillaris* leaves	[70, 71, 93]
	Trametes	*T. robiniophila* Murr	*Radix isatidis*	[104]
	Grifola	*G. frondosa*	*Rhizoma gastrodiae*	[105]
	Coprinus	*C. comatus*	*Sophora flavescens*	[106]

## PROBIOTIC FERMENTATION TECHNIQUES FOR CHM

**Figure 3:**
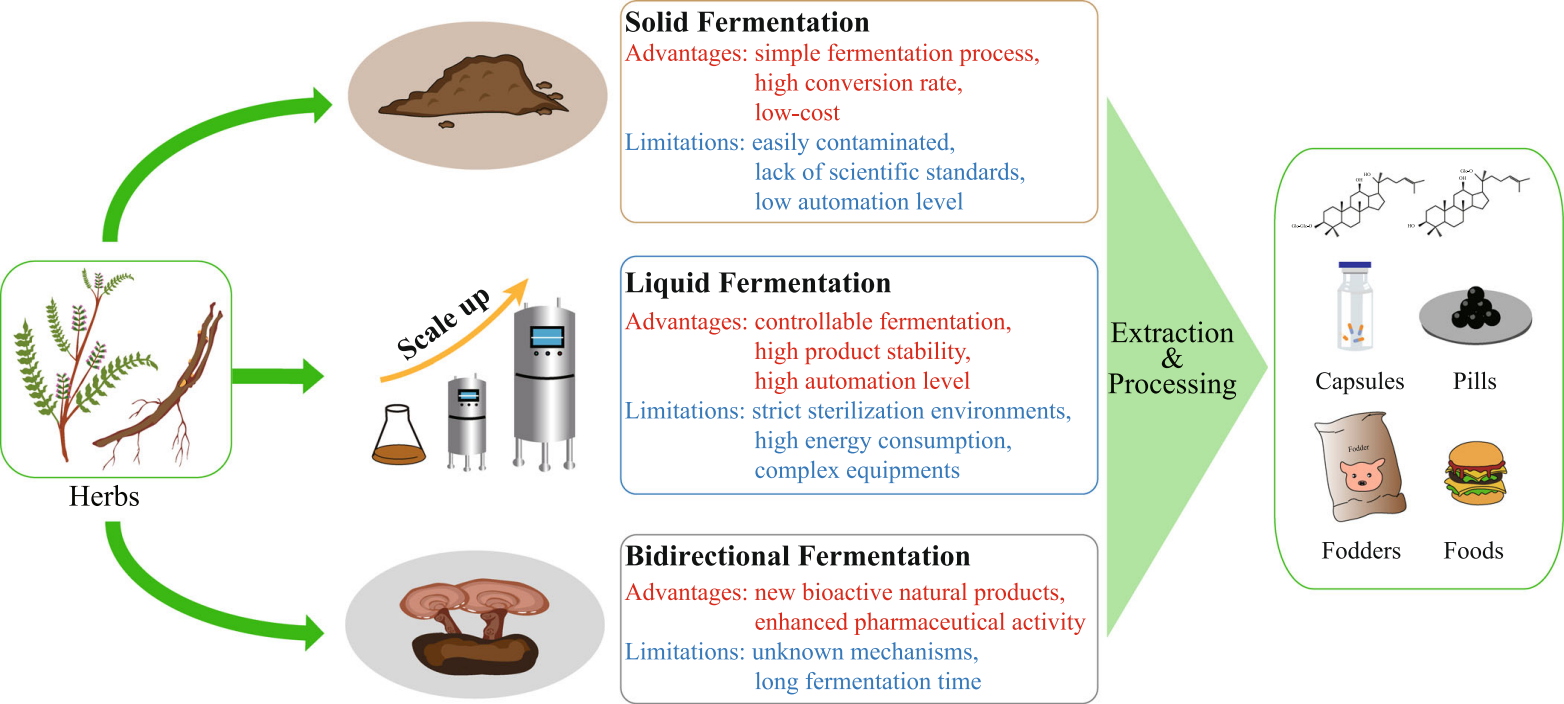
Different probiotic CHM fermentation strategies and their characterization. The liquid, solid, and bidirectional fermentation were used for CHM fermentation. After extraction and purification, the final products of CHM fermentation could be used as drugs, fodders, and foods. CHM, Chinese herbal medicines.

Traditional CHM fermentation technique is solid‐state fermentation, which uses wild‐type microorganisms in the environments to complete the fermentation process without accurate control of ambient temperature and humidity. The fermentation endpoint of solid‐state fermentation is often determined by individual experience. Therefore, the efficacy, safety, and stability of traditional fermented CHM are not stable, and this might due to insufficient strain purity, uncontrollable fermentation conditions, and lack of standardized fermentation process and appropriate monitoring indicators. Compared with traditional fermentation technique, modern fermentation technology integrates microbial ecology, fermentation engineering, and bioengineering, leading to new CHM fermentation techniques [15]. On the basis of fermentation forms, modern fermentation techniques can be divided into solid fermentation, liquid fermentation, and bidirectional fermentation with medicinal fungi (Figure 3).

### Solid fermentation

Solid fermentation uses one or several probiotic strains to ferment CHM biomass under low‐moisture or almost no free‐water conditions [17]. The solid fermentation system is naturally open, and sterilization of the substrates is not necessary (Figure 3). Moreover, solid fermentation generates small amounts of wastewater [107]. The cost of solid fermentation is low, and the procedure is relatively simple [108]. Solid fermentation has the advantages of high conversion rate and high yield. The solid fermentation converts 20%–30% CHM substrates to novel products, while the transformation efficiency of liquid fermentation is only about 5% [86]. However, solid fermentation has some limitations, including frequent contamination by miscellaneous bacteria due to the open fermentation system, slow fermentation rate, lack of scientific standards for fermentation endpoint and quality control, and low automation level [109].

In addition to the traditional starter‐making technology, a variety of novel solid probiotic fermentation systems for CHM have been developed [110]. After solid fermentation of ginseng seeds with Bacillus, Lactobacillus, and Pediococcus strains, respectively, the contents of total sugars, acidic polysaccharides, and phenolic compounds were higher than that of the nonfermented control [89]. Moreover, the antioxidant activity of ginseng seeds improved after probiotic solid fermentation [89]. Solid fermentation of *Astragalus membranaceus* with *L. plantarum* and *Enterococcus faecium* greatly improves the production of health‐promoting biological compounds, including polysaccharides, total saponins, and flavonoids [52].

### Liquid fermentation

Liquid fermentation, also known as liquid‐submerged fermentation, is derived from the antibiotics production process [111]. Liquid fermentation technique inoculates the activated microorganisms into the medium composed with CHM extracts and proper microbial nutrients (Figure 3). The fermentation process was implemented under suitable temperature and pH value. Compared with solid fermentation, liquid fermentation has the advantages of high product stability, quantified production conditions, and high automation level. Moreover, liquid fermentation can be efficiently applied in large‐scale CHM fermentation [15]. Liquid fermentation requires strict sterilization environments, and the fermentation process is high energy consumption; moreover, the equipment is complex [15]. Thus, it is necessary to optimize the liquid fermentation process, especially fermentation devices and conditions, to improve active ingredient conversion rate and reduce pollution.

Some probiotic liquid fermentation strategies for CHM were applied. For example, liquid fermentation of hydroponic ginseng with *Lactococcus lactis* KC24 increased the antioxidant activity of ginseng [98]. Red ginseng fermented with *L. paracasei* and *Bifidobacterium longum* could efficiently alleviate ovalbumin‐induced inflammation in mice [58]. The hematopoietic activity of deer antler increased after the liquid fermentation with *B. subtilis* [99].

### Bidirectional fermentation with medicinal fungi

Bidirectional fermentation with medicinal fungi includes liquid fermentation and solid fermentation; the former is the combination of basic culture medium, CHM extracts, and fungi in closed environment, while the latter is the combination of CHM and fungi in open environments. Bidirectional solid fermentation was established in the 1980s [112]. It is a new Chinese herbal fermentation technique that CHM substrates are fermented by medicinal fungi (Figure 3). During bidirectional fermentation, CHM substrates provide the nutrients for medicinal fungi growth, and the fungal fermentation increases the bioactive natural product composition of the CHM substrates [17]. Bidirectional fermentation could produce a large number of new bioactive fermentation metabolites. Insight into the fermentation process and fungal enzymatic systems would give clues to the bidirectional fermentation mechanisms [113].

Fresh ginseng fermented with *G. lucidum* mycelium in solid‐state culture could enhance its immunomodulatory activity [59]. The solid‐state bidirectional fermentation of *A. capillaris* leaves with *G. lucidum* enhanced the anti‐inflammatory effects in a mice model with atopic dermatitis [93]. Products of *Trametes robiniophila* Murr fermented with *Radix isatidis* strongly inhibited the cell proliferation of breast cancer cells [104]. Compared with the control, when *Ginkgo biloba* leaves were fermented with *G. lucidum* by bidirectional liquid fermentation, the yield of polysaccharides, triterpenes, and total flavonoids increased by 2.38, 1.96, and 2.10 times, respectively, which leads to higher antioxidation activity of the fermentation products [114]. However, the bidirectional fermentation rate is slow, and the application of the cutting‐edge genetic engineering tools is limited [115].

## PROBIOTIC FERMENTATION MODES FOR CHM FERMENTATION

### Single probiotic strain fermentation

Single probiotic strain fermentation is the most commonly used fermentation mode, and the probiotic fermentation modifies the structure of specific substrates by enzymatic catalysis [116, 117]. The strains of Lactobacillus, Bifidobacterium, Bacillus, and some medicinal fungi are often used for single‐strain CHM fermentation. The metabolites of *P. ginseng* fermented with *Lactobacillus fermentum* can treat antibiotic‐associated diarrhea symptoms and colon inflammation [21]. Moreover, the fermentation metabolites could transfer the gut microbiota disturbances to healthy state in rat [21]. Fermentation of *Artemisia princeps* Pampanini with *L. plantarum* SN13T increased the amounts of bioactive compounds of catechol and seco‐tanapartholide C [50]. The fermentation of red ginseng with *B. animalis* subsp. *lactis* LT 19‐2 isolated from the feces of infants could enhance immunomodulatory function of red ginseng [73]. Fermentation of *Cynanchi atrati* Radix with Lactobacillus increased the anti‐melanin activity [118]. As single probiotic strain fermentation can increase the performance of CHM, more probiotics might be applied in future CHM fermentation.

### Multispecies fermentation

Compared with single‐strain fermentation, multispecies fermentation can provide diverse and redundant enzymatic systems. Multispecies fermentation has potential to improve the utilization rate and increase biotransformation efficiency of CHM [17]. Synthetic microbiota with bacteria, fungi, or bacteria‐fungi has been used for CHM fermentation. *Salvia miltiorrhiza* Bunge (Danshen) fermented by *L. rhamnosus* (F‐B4‐1) and *B. subtilis Natto* (F‐A7‐1) relieved dextran sulfate sodium‐induced ulcerative colitis in mice more effectively than raw Danshen [85]. Fermented white ginseng roots with *B. subtilis* and *Trichoderma reesei* enhanced the biotransformation yield from ginsenosides to rare ginsenosides, for these two species have nonsynchronous cell growth and different metabolic pathways [100]. The coimmobilized edible *Aspergillus niger* and yeast produced 11‐fold resveratrol from polydatin in *Polygonum cuspidatum* roots than that of the untreated sample [53]. At present, few multispecies fermentations of CHM are available, which might due to the complex control and modulation of multispecies fermentation. In the future, optimization of multispecies fermentation process and recovery of the underlying mechanisms are of great value to CHM fermentation.

## APPLICATION OF MICROBIOME AND SYNTHETIC BIOLOGY STRATEGIES FOR EFFICIENT PROBIOTIC FERMENTATION OF CHM

**Figure 4:**
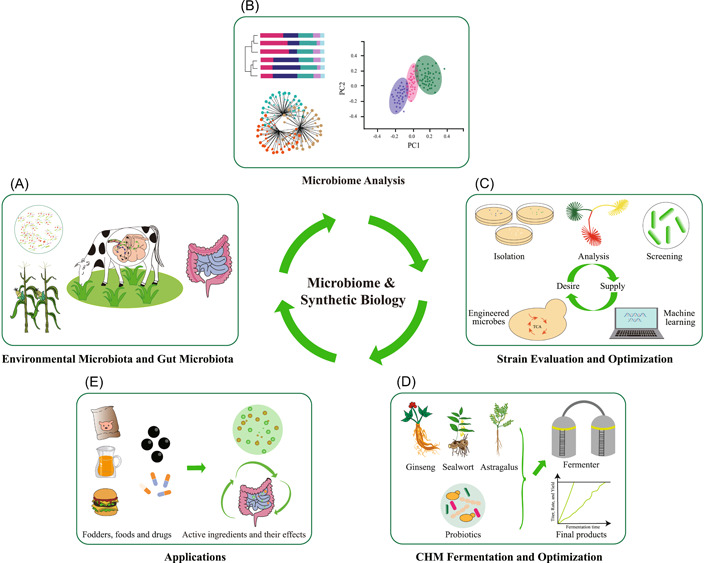
Application of microbiome and synthetic biology strategies for efficient probiotic CHM fermentation. (A) Environmental microbiota, and human and animal gut microbiota are potential microbial sources for CHM fermentation. (B) Environmental microbiota and gut microbiota can be analyzed by microbiome strategies, and screened for probiotics. (C) Efficient probiotics can be isolated, analyzed, and screened for CHM fermentation. Moreover, the machine learning and metabolic engineering technologies can provide further efficient enzymes or microbes for CHM fermentation. (D) Fermentation process can be optimized, which can lead to the production of bioactive ingredients with high yield. (E) The obtained CHM fermentation products can be applied in foods, animal feeds, drugs, or other industries. The active ingredients would produce beneficial effects for humans and animals. CHM, Chinese herbal medicines.

CHM fermentation has the advantages of increasing pharmacological activity, reducing toxicity, and producing new bioactive ingredients. Understanding the underlying mechanisms of CHM fermentation lays the foundation for the optimization of probiotic fermentation [119]. High‐quality and safe fermentation strains are the basis and keystone for CHM fermentation [120]. Currently, most probiotic strains used for CHM fermentation derived from fermented dairy products and the animal fecal microbiota [121, 122]. The probiotic types are very limited, which are mainly assigned to Lactobacillus, Bifidobacterium, Bacillus, and yeasts [123]. With the development of synthetic microbiology technologies, efficient and affordable high‐throughput sequencing technologies help recover probiotics in diverse environments (Figure 4A,B) [124, 125, 126]. The microbiome strategies have been applied to reveal the microbial variations during the manufacturing process of Fu brick tea, the spontaneous fermentation periods of light‐flavor *Baijiu*, and the fermentation of Huafeng Dan Yaomu [127, 128, 129]. Though most microorganisms are uncultured, the new developed culturomics provide tools to isolate and screen proper probiotics for CHM fermentation (Figure 4C) [130, 131, 132].

The efficient hydrolase and other CHM biomass hydrolysis enzymes, especially lignocellulases, transform CHM substrates to bioactive natural products and generate/produce prebiotics from lignocellulose (Figures 1C and 4D,E). Thus, recovery and characterization of efficient enzymes for CHM fermentation are essential. For example, ginsenosides are believed to be the primary beneficial components of ginseng, but its oral bioavailability is low. Ginsenoside transformed by human gut microbiota could increase biological activity and bioavailability in vivo [133]. The biotransformation mechanism of human gut microbiota is hydrolysis of sugar moieties of ginsenosides by *β*‐glucosidase derived from gut microbiota to produce rare ginsenosides (Figure 2C) [133]. An *A. niger* XD101 strain could transform Rb1 to easily absorbed ginsenoside CK by its extracellular *β*‐glucosidase [134]. In addition, a variety of probiotics with high *β*‐glucosidase activity have been screened for *P. ginseng* fermentation, including *B. lactis* Bi‐07 [75], *L. rhamnosus* HN001 [75], and *Lentilactobacillus buchneri* URN103L [134]. Baicalin (baicalein 7‐*O*‐*β*‐d‐glucuronide) is one of the major flavonoids in *Scutellaria baicalensis*. Baicalein, the aglycone of baicalin, is easier to be absorbed and more effective than baicalin, but the content of baicalein in *S. baicalensis* is relatively low. *Lactobacillus brevis* subsp. *coagulans* can convert baicalin to baicalein using its *β*‐glucuronidase [135]. More than 90,000 genes/gene fragments encoding for carbohydrate‐active enzymes were recovered from diverse cellulolytic microorganisms [136]. Further enzymatic characterization identified some xylanase and pectinolytic enzymes [137, 138], suggesting that efficient hydrolase for CHM fermentation could be recovered from natural environments using microbiome strategies.

Synthetic biology provides valuable tools for the optimization of enzymes and strains with efficient CHM fermentation ability. The protein engineering and metabolic engineering based on machine learning can improve hydrolase activities and other performances, which would provide efficient engineered enzymes/microbes for CHM fermentation (Figure 4C) [139]. The CHM bioactive natural product yield can be improved by optimization of synthetic fermentation microbiota and fermentation parameters (Figure 4D). Fermentation with *B. subtilis* and *T. reesei* promoted biotransformation efficiency of ginsenosides in white ginseng roots, and the inoculation proportion of *B. subtilis* and *T. reesei* at 1:4 resulted in the highest rare ginsenoside yield [100]. Pretreatment of *P. cuspidatum* root with immobilized *β*‐glucosidases could improve the conversion of polydatin to resveratrol at proper fermentation environment [140]. Designing and building synthetic microbiota with wild‐type probiotics, and optimizing fermentation parameters, including pH value, temperature, and incubation time, could improve the yield of bioactive natural products generated by CHM fermentation [141] (Figure 4D). Production of terpenoids, lipids and other plant natural products by engineered yeasts has been achieved [142, 143, 144, 145], and synthetic biology could design and reprogram of microorganisms to *de novo* produce various bioactive natural products [139, 146, 147, 148, 149, 150]. In the future, production of CHM bioactive natural products by synthetic biology technology might be an alternative strategy for probiotic CHM fermentation, and the products can be further applied in foods, animal feed, and other industries [151].

## FUTURE PERSPECTIVES

The contents of many bioactive ingredients in CHM are low, and some CHM components are toxic. Probiotic fermentation of CHM can generate easily absorbed bioactive compounds and reduce toxicities. Therefore, discovering efficient and safe probiotic strains and developing novel fermentation strategies for CHM fermentation are of great interest. Insights into the generation pathway of active ingredients could accelerate to screen efficient enzymes and probiotics for CHM fermentation. Optimization of fermentation equipments and parameters are necessary to obtain high titer, rate, and yield of CHM bioactive products. Although probiotics are safe for the human body, the products of probiotic fermentation should accept a comprehensive and scientific safety evaluation. Thus, CHM fermentation standards should be drafted and optimized before application. Probiotic fermentation of CHM would not only offer opportunities to recover underlying mechanisms for bioactive natural product generation, but also provide healthy products for humans and animals. In the future, the development of synthetic biology would lead to the production of CHM bioactive natural products with efficient microbial cell factories.

## AUTHOR CONTRIBUTIONS

Yongjun Wei, Lingbo Qu, and Yulong Yin conceived the study. Xiaoling Zhang, Qin Miao, Yongjun Wei, and Chenxue Pan drafted and revised the manuscript. Xiaoling Zhang, Qin Miao, and Yongjun Wei prepared the figures and tables. Jia Yin, Leli Wang, Lingbo Qu, and Yulong Yin revised the manuscript. Yongjun Wei and Xiaoling Zhang designed the study. All the authors read and approved the manuscript.

## CONFLICT OF INTEREST STATEMENT

The authors declare no conflict of interest.

## Data Availability

Supplementary materials (figures, tables, scripts, graphical abstract, slides, videos, Chinese translated version, and update materials) may be found in the online DOI or iMeta Science http://www.imeta.science/.
